# Supplementary material for: CAR T cells expressing a bacterial virulence factor trigger potent bystander antitumour responses in solid cancers
Source: Nat Biomed Eng. 2022 Apr 4;6(7):830–41. doi: 10.1038/s41551-022-00875-5 (PMC9288934; doi:10.1038/s41551-022-00875-5)
Supplement: Supplementary file 1 — Supplementary figures. [file 41551_2022_875_MOESM1_ESM.pdf]

nature portfolio

https://doi.org/10.1038/s41551-022-00875-5

Supplementary information

CAR T cells expressing a bacterial virulence factor trigger potent bystander antitumour responses in solid cancers

In the format provided by the authors and unedited

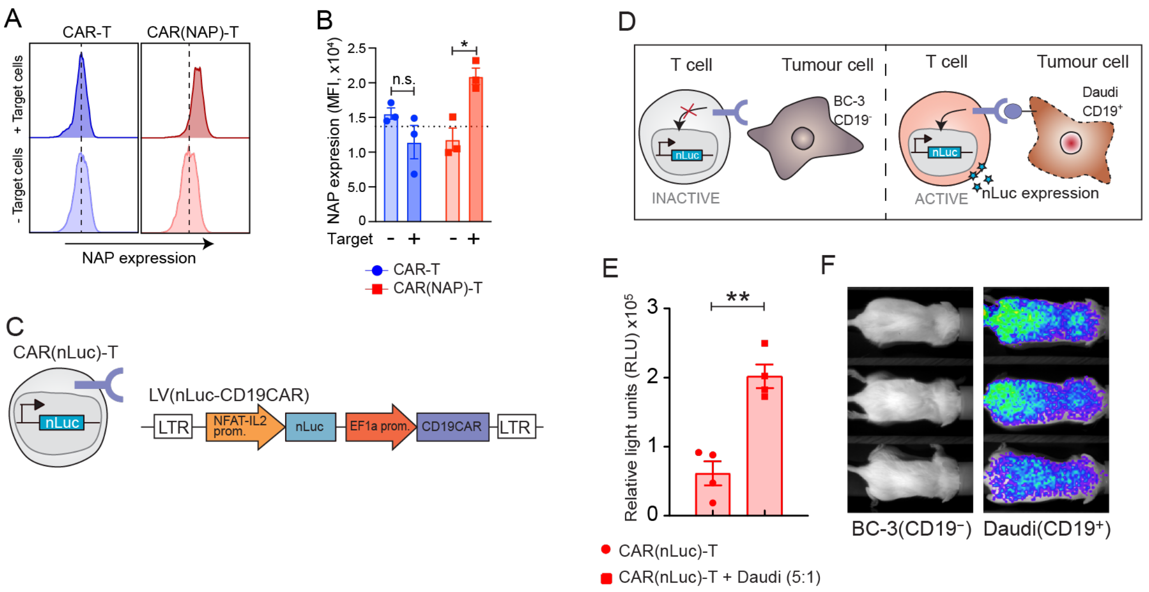

Supplementary Fig. 1 I The inducible NFAT-IL2 promoter is activated upon CAR engagement with target antigen *in vitro* and *in vivo*. (A) Representative histogram and (B) Mean fluorescent intensity (MFI) of NAP expression in CAR-positive T cells upon target cell recognition. Dotted line: MFI of isotype staining. (C) Lentiviral construct used for human T-cell engineering with nano-luciferase (nLuc) as reporter gene. (D) Illustration of NFAT-IL2 promoter activation and transgene (nLuc) expression upon target cell recognition. (E) The amount of nLuc released by human CAR(Luc) T cells after 24-h *in vitro* incubation alone or in co-culture with Daudi target cells ($CD19^+$) (**: P<0.01). (F) nLuc expression by CAR(nLuc) T cells, monitored by *in vivo* luminescence imaging, 3 d after intravenous injection into NOD-SCID mice, together with Daudi ($CD19^+$) or BC-3 ($CD19^-$) target cells, at a 2:1 ratio. Error bars represent SEM. Precise *P*-values are reported in Supplementary Table 3.

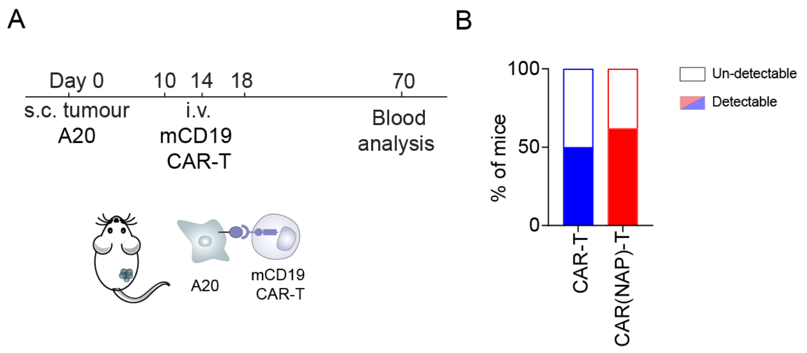

Supplementary Fig. 2 l The persistence of CAR(NAP) T cells is comparable to that of CAR T cells in the blood of treated mice. (A) Experimental design and treatment schedule for mice with subcutaneous A20 tumours. (B) Proportion of mice with qRT-PCR detectable CAR-engineered T cells in the blood 70 d after tumour cell implantation. (CAR T cells: n=6; CAR(NAP) T cells: n=8).

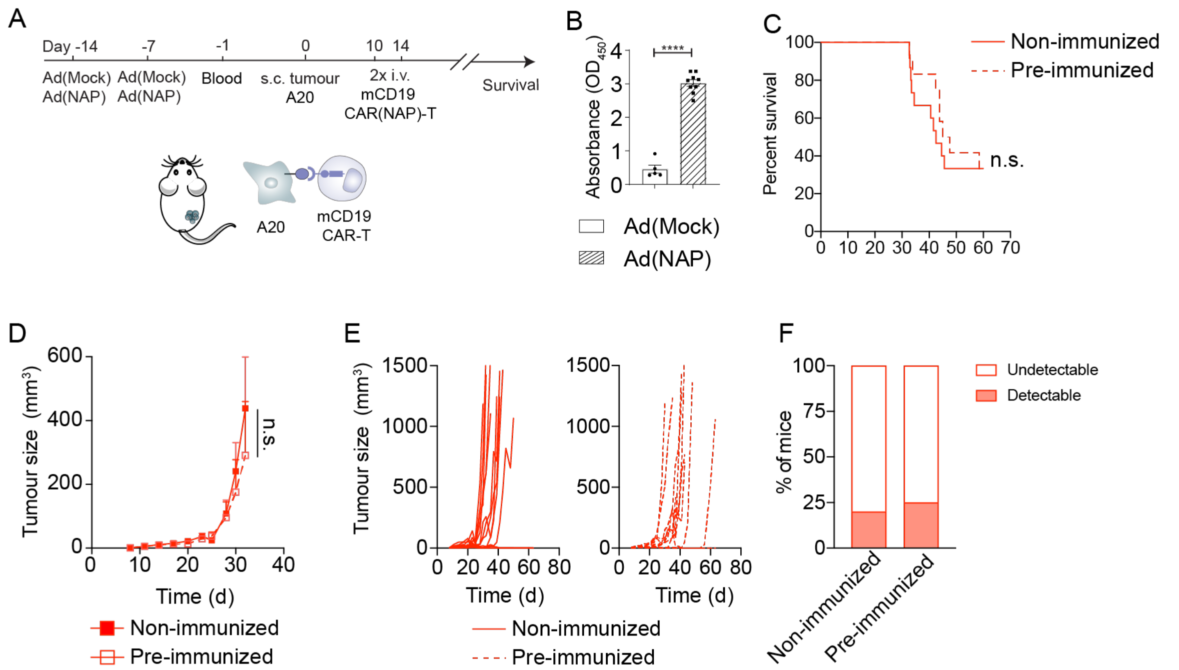

Supplementary Fig. 3 l Pre-existing anti-NAP antibodies in mice does not affect the cytotoxic capacity or persistence of CAR(NAP) T cells. (A) Experimental design with immunization before subcutaneous A20 implantation and treatment (2x i.v.) with CAR-engineered T cells. (B) Anti-NAP antibody level in the blood of mice after prime/boost with Ad(Mock) or Ad(NAP). (C) Mouse survival (Kaplan-Meier curve) after treatment, (D) Mean tumour size until the first mouse had to be sacrificed and (E) individual mouse tumour size after treatment. (F) Proportion of tumour-free mice with qRT-PCR detectable CAR-engineered T cells in the blood on day 60 (Pre-immunized: n=4 and Non-immunized: n=5). All experiments were repeated and data were pooled. Error bars represent SEM (n.s.: no significance; ****: $P<0.0001$). Precise P-values are reported in Supplementary Table 3.

A

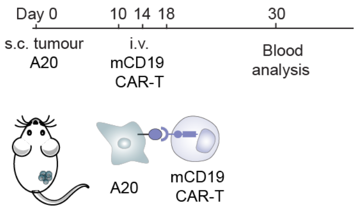

B

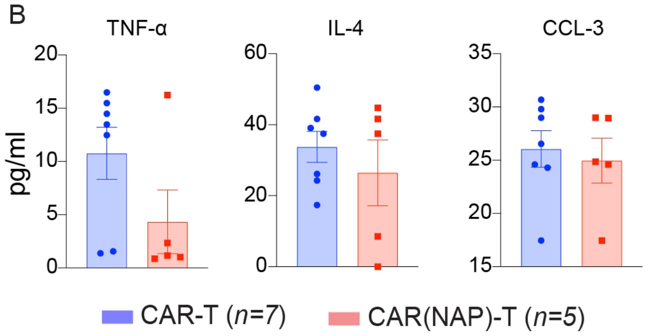

C Non-detectable cytokines

	IFN-γ	IL-10	CCL4	IFN-α	CXCL10	IL-6	VEGF	CCL2	GM-CSF
CAR T-cells	<0.24	<0.24	<4.26	<0.24	<0.94	<12.05	<0.24	<0.24	<2.54
CAR(NAP) T-cells	<0.24	<0.24	<4.26	<0.24	<0.94	<12.05	<0.24	<0.24	<2.54

Supplementary Fig. 4 I CAR(NAP)-T treatment does not induce systemic cytokine release. (A) Experimental design and treatment schedule for mice with subcutaneous A20 tumours. Cytokines in the blood were analysed on day 30. (B) Cytokine levels (TNF-α, IL-4 and CCL-3) measured by LEGENDplex and (C) Cytokines with levels below detection limit. Error bars represent SEM.

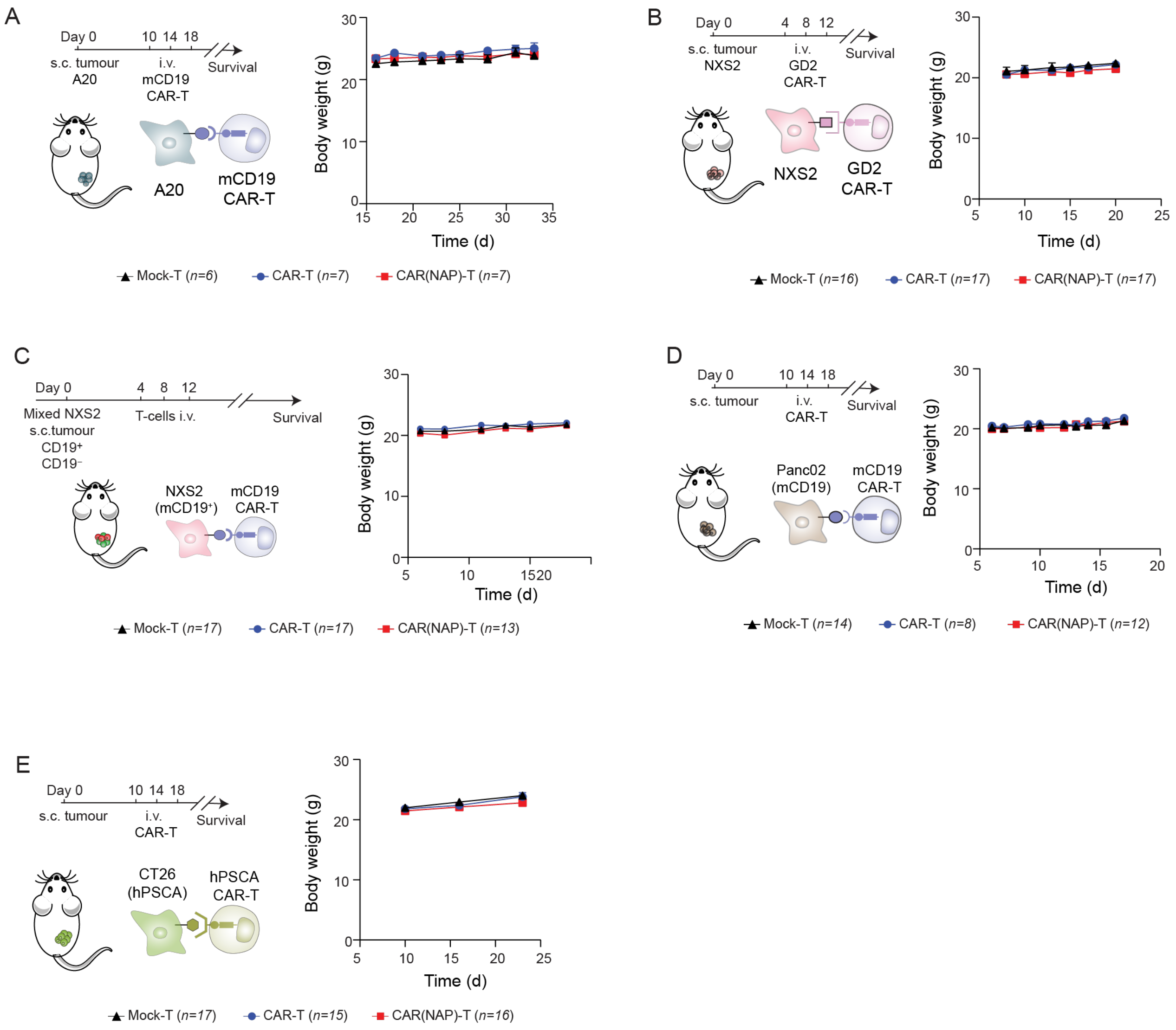

Supplementary Fig. 5 I CAR(NAP) T cell treatment does not alter the body weight of mice. Body weight of mice after treatment in the (A) A20, (B) NXS2, (C) NXS2/NXS2-mCD19 mixed tumour, (D) Panc02-mCD19 and (E) CT26-hPSCA models. Error bars represent SEM.

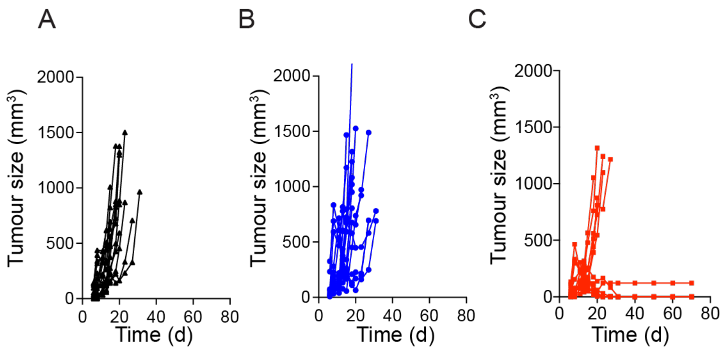

Supplementary Fig. 6 l Tumour sizes in individual mice in the NXS2/NXS2-CD19 mixed tumour models after treatment. Tumour size after treatment with (A) Mock T cells, (B) CAR T cells, and (C) CAR(NAP) T cells.

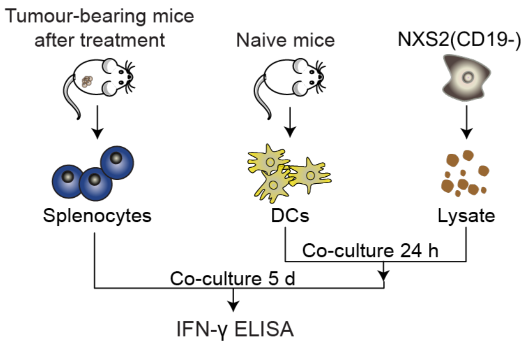

Supplementary Fig. 7 l Illustration of the re-call assay to assess bystander T cell immunity. (A) Illustration of the re-call assay mentioned in Fig. 2G for evaluating systemic bystander T-cell activation and epitope spread, wherein the splenocytes in each treatment group were harvested and used.

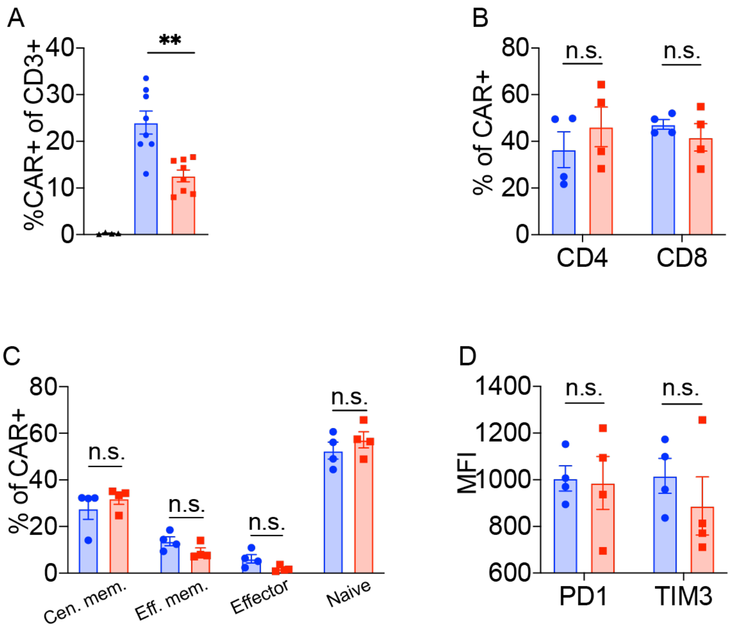

Supplementary Fig. 8 l Phenotypic characterization of CAR-engineered T cells. (A) Transduction efficiency of CAR T cells and CAR(NAP) T cells. (B) CD4 and CD8 T cell compartment in CAR+ T cells after transduction. (C) Central (Cen.) and effector (Eff.) memory (mem.) cell, Effector cell, and Naïve cell composition in the CAR+ population after transduction. (D) Mean fluorescent intensity (MFI) of PD1 and TIM3 on CAR+ T cells. Data are pooled from each experiment and each dot represents one donor. Error bars represent SEM (n.s.: no statistical significance, **: *P*<0.01). Precise *P*-values are reported in Supplementary Table 3.

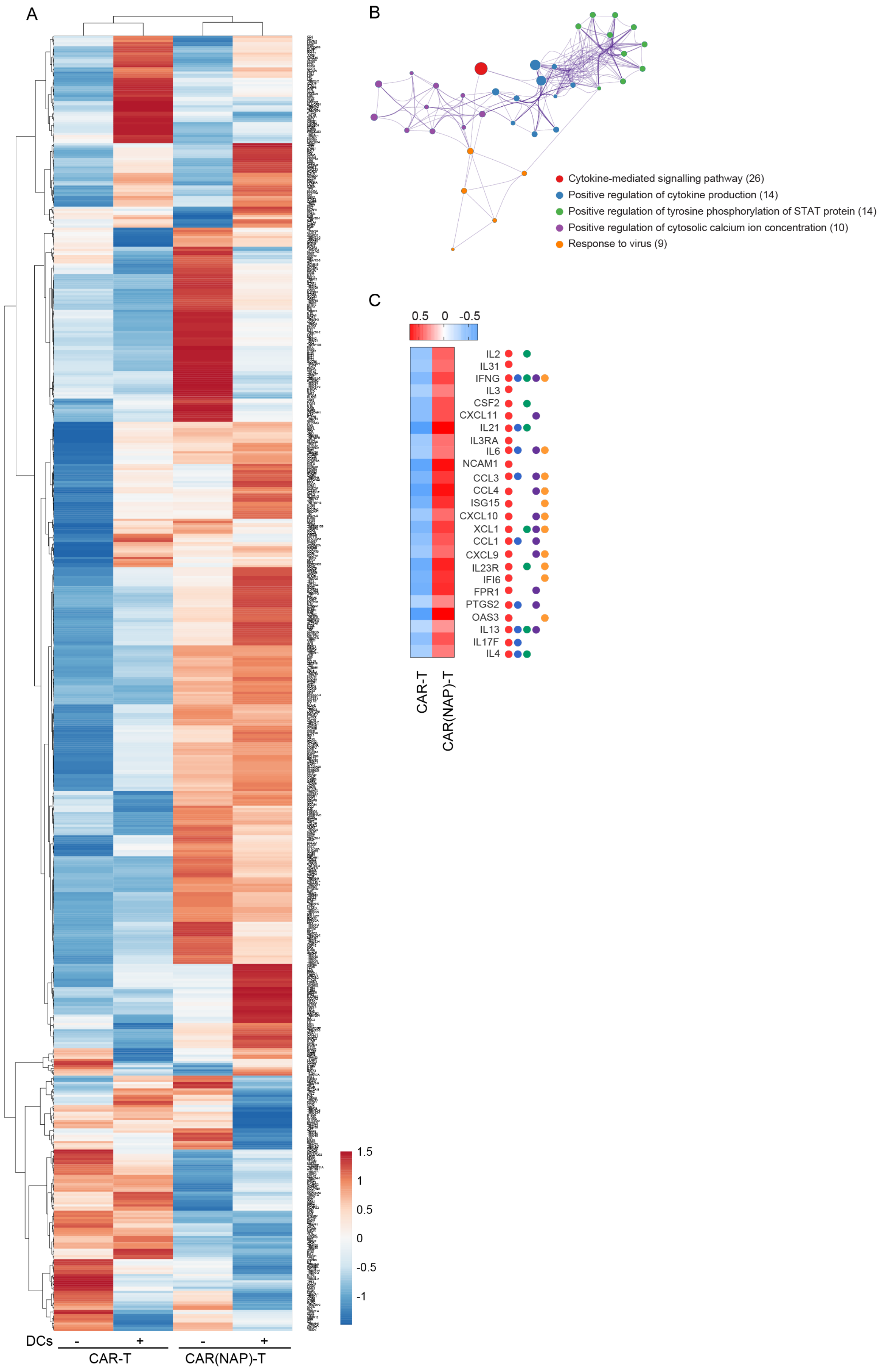
A
DCs
-
+
-
+
CAR-T
CAR(NAP)-T
1.5
1
0.5
0
-0.5
-1
B
Cytokine-mediated signalling pathway (26)
Positive regulation of cytokine production (14)
Positive regulation of tyrosine phosphorylation of STAT protein (14)
Positive regulation of cytosolic calcium ion concentration (10)
Response to virus (9)
C
0.5
0
-0.5
IL2
IL31
IFNG
IL3
CSF2
CXCL11
IL21
IL3RA
IL6
NCAM1
CCL3
CCL4
ISG15
CXCL10
XCL1
CCL1
CXCL9
IL23R
IFI6
FPR1
PTGS2
OAS3
IL13
IL17F
IL4
CAR-T
CAR(NAP)-T

**Supplementary Fig. 9 | **Gene expression profile of human CAR T cells and CAR(NAP) T cells upon target activation with/without feedback help from DCs. (A) Gene expression in CAR T cells and CAR(NAP) T cells, in the absence (–) or presence (+) of autologous DCs, after *in vitro* activation via target antigen recognition. Mean values from three different donors are presented. (B) GO term enrichment network of uniquely upregulated genes in human CAR(NAP) T cells according to Metascape analysis of the NanoString gene expression data. Numbers in bracket are -log10(*P*). (C) Heatmap of gene expression of the genes in different GO term pathways and classification are color-coded according to B.
