# Supplementary material for: CAR T cells expressing a bacterial virulence factor trigger potent bystander antitumour responses in solid cancers
Source: Nat Biomed Eng. 2022 Apr 4;6(7):830–41. doi: 10.1038/s41551-022-00875-5 (PMC9288934; doi:10.1038/s41551-022-00875-5)
Supplement: Supplementary file 2 — Reporting Summary [file 41551_2022_875_MOESM2_ESM.pdf]

nature portfolio

Corresponding author(s): Di Yu and Magnus Essand

Last updated by author(s): Feb 23, 2022

Reporting Summary

Nature Portfolio wishes to improve the reproducibility of the work that we publish. This form provides structure for consistency and transparency in reporting. For further information on Nature Portfolio policies, see our Editorial Policies and the Editorial Policy Checklist.

Statistics

For all statistical analyses, confirm that the following items are present in the figure legend, table legend, main text, or Methods section.

n/a	Confirmed	
☐	☒	The exact sample size (n) for each experimental group/condition, given as a discrete number and unit of measurement
☐	☒	A statement on whether measurements were taken from distinct samples or whether the same sample was measured repeatedly
☐	☒	The statistical test(s) used AND whether they are one- or two-sided<br>*Only common tests should be described solely by name; describe more complex techniques in the Methods section.*
☐	☒	A description of all covariates tested
☐	☒	A description of any assumptions or corrections, such as tests of normality and adjustment for multiple comparisons
☐	☒	A full description of the statistical parameters including central tendency (e.g. means) or other basic estimates (e.g. regression coefficient) AND variation (e.g. standard deviation) or associated estimates of uncertainty (e.g. confidence intervals)
☐	☒	For null hypothesis testing, the test statistic (e.g. F, t, r) with confidence intervals, effect sizes, degrees of freedom and P value noted<br>*Give P values as exact values whenever suitable.*
☒	☐	For Bayesian analysis, information on the choice of priors and Markov chain Monte Carlo settings
☒	☐	For hierarchical and complex designs, identification of the appropriate level for tests and full reporting of outcomes
☒	☐	Estimates of effect sizes (e.g. Cohen's d, Pearson's r), indicating how they were calculated

Our web collection on statistics for biologists contains articles on many of the points above.

Software and code

Policy information about availability of computer code

Data collection	Flow cytometry: BD Diva; CytExpert; BD FACSChorus; BD LSRFortessa<br>Microscopy: Zeiss AxioImager<br>Protein multiplexing imaging: BioRad Imager
Data analysis	Graphpad v6.07 - v9.3.1;<br>Nanostring: nSolver 4.0<br>GO analysis: Metascape<br>Flow: FlowJo v10.2 - v10.8.1

For manuscripts utilizing custom algorithms or software that are central to the research but not yet described in published literature, software must be made available to editors and reviewers. We strongly encourage code deposition in a community repository (e.g. GitHub). See the Nature Portfolio guidelines for submitting code & software for further information.

Data

Policy information about availability of data

All manuscripts must include a data availability statement. This statement should provide the following information, where applicable:

- Accession codes, unique identifiers, or web links for publicly available datasets
- A description of any restrictions on data availability
- For clinical datasets or third party data, please ensure that the statement adheres to our policy

The main data supporting the results in this study are available within the paper and its Supplementary Information. Source data for the tumour-growth curves are provided with this paper. All data generated in this study are available from the corresponding authors on reasonable request.

Field-specific reporting

Please select the one below that is the best fit for your research. If you are not sure, read the appropriate sections before making your selection.

☒ Life sciences ☐ Behavioural & social sciences ☐ Ecological, evolutionary & environmental sciences

For a reference copy of the document with all sections, see nature.com/documents/nr-reporting-summary-flat.pdf

Life sciences study design

All studies must disclose on these points even when the disclosure is negative.

Sample size	No sample-size calculations were performed. Sample size was based on previous experience and pilot studies, and each experiment was repeated at least once.
Data exclusions	Mice injected with tumour cells and that did not show tumour growth were not included. Mice that died for unknown reasons were excluded. No data were excluded from the analyses.
Replication	The animal studies were performed at least twice, and the data were pooled. In vitro data were generated from at least 3 donors, as detailed in the relevant figure captions.
Randomization	All groups of mice were age-matched. The mice were randomized prior to treatment, without knowledge of tumor burden.
Blinding	Blinding was irrelevant to the study. All mice experiments were carried out by researchers who also prepared the CAR-T cells before administration into mice.

Reporting for specific materials, systems and methods

We require information from authors about some types of materials, experimental systems and methods used in many studies. Here, indicate whether each material, system or method listed is relevant to your study. If you are not sure if a list item applies to your research, read the appropriate section before selecting a response.

Materials & experimental systems

n/a	Involved in the study	
☐	☒	Antibodies
☐	☒	Eukaryotic cell lines
☒	☐	Palaeontology and archaeology
☐	☒	Animals and other organisms
☐	☒	Human research participants
☒	☐	Clinical data
☒	☐	Dual use research of concern

Methods

n/a	Involved in the study	
☒	☐	ChIP-seq
☐	☒	Flow cytometry
☒	☐	MRI-based neuroimaging

Antibodies

Antibodies used	All antibodies are listed in Supplementary Table 1.
Validation	Antibody validation was performed by the relevant supplier.

Eukaryotic cell lines

Policy information about cell lines

Cell line source(s)	Daudi, BC-3, A20 and CT26 cells were procured from ATCC; NXS2 was a gift from Holger N. Lode, University of Greifswald; Panc02 was a gift from Rainer Heuchel, Karolinska Institute. Human PBMCs (source of T cells) were freshly isolated from buffycoat. Mouse splenocytes (source of T cells) were freshly isolated from mouse spleens. The Gryphon retroviral packaging cell line was procured from Allele Biotechnology, San Diego, CA. The 293T lentiviral/retroviral vector packaging cell line was procured from ATCC.
Authentication	The cell lines purchased from ATCC were not authenticated. Murine cell lines from C57bl/6 were confirmed with the strain, but cannot be authenticated by STR.
Mycoplasma contamination	All cell lines tested negative for mycoplasma, using a Lonza kit.

Commonly misidentified lines (See ICLAC register)	No commonly misidentified cell lines were used (according to ICLAC version 11; checked on 07 February 2022).

Animals and other organisms

Policy information about studies involving animals; ARRIVE guidelines recommended for reporting animal research

Laboratory animals	6–8-week-old female C57Bl/6NRj and Balb/c from Taconic Denmark, and 6–8-week old female A/J mice from Envigo, the Netherlands.
Wild animals	The study did not involve wild animals.
Field-collected samples	The study did not involve samples collected from the field.
Ethics oversight	The Uppsala Research Animal Ethics Committee approved all animal studies (N164/15; N185/16; 5.8.18-19434/2019).

Note that full information on the approval of the study protocol must also be provided in the manuscript.

Human research participants

Policy information about studies involving human research participants

Population characteristics	The human buffy coats obtained from healthy blood donors had been anonymized.
Recruitment	Peripheral blood mononuclear cells were isolated by Ficoll-Paque (GE Healthcare Life Science, Uppsala, Sweden) from fresh buffy coats of healthy anonymized donors, collected at the Blood Centre at Uppsala University Hospital.
Ethics oversight	Because the samples had been anonymized, an ethical permit was not required.

Note that full information on the approval of the study protocol must also be provided in the manuscript.

Flow Cytometry

Plots

Confirm that:

☐ The axis labels state the marker and fluorochrome used (e.g. CD4-FITC).

☐ The axis scales are clearly visible. Include numbers along axes only for bottom left plot of group (a 'group' is an analysis of identical markers).

☐ All plots are contour plots with outliers or pseudocolor plots.

☒ A numerical value for number of cells or percentage (with statistics) is provided.

Methodology

Sample preparation	For the cell-culture experiments: suspension cells were harvested directly, washed with PBS and resuspended in ca. 200 µL of PBS containing the indicated antibody mixture (prepared as a master-mix solution).<br><br>For samples with intracellular-staining steps: cells were first stained with surface markers, then permeabilized with BDperm buffer (BD Biosciences) and additionally stained with antibodies targeting an intracellular marker (such as IL-2).<br><br>For samples with intra-nucleus staining steps: cells were first stained with surface markers, then permeabilized with True-Nuclear Transcription Factor Buffer set (Biolegend), then washed, and stained with additional antibodies (such as T-bet).<br><br>For NAP-expression detection: cells were treated with Brefeldin A (BD GolgiPlug, BD BioSciences) before staining.<br><br>For the in vivo experiments: The tumour samples were collected and enzymatically digested (Liberase, Roche) into single-cell suspensions. CD45+ cells were bead-isolated (Miltenyi Biotec) and stained with the appropriate antibody mixture.
Instrument	BD Canto II; BD Melody; CytoFLEX S; CytoFLEX XL.
Software	BD Diva; CytExpert; BD FACSChorus.
Cell population abundance	At least 10,000 cells were recorded for CD3+ for T cells; at least 1x10^6 for alive CD45+ cells were recorded when analysing tumour-infiltrating cells; otherwise, at least 10,000 cells in the FSC/SSC gate were recorded.
Gating strategy	Preliminary FSC-A/SSC-A gates were used on morphology and FSC-A/FSC-H for singlets, then followed by Zombie Aqua live/dead gating. Further gating for each population are detailed in Methods and in the relevant figure captions.

☒ Tick this box to confirm that a figure exemplifying the gating strategy is provided in the Supplementary Information.
